# Activation of NLRP3 Inflammasome and Onset of Alzheimer’s Disease

**DOI:** 10.3389/fimmu.2021.701282

**Published:** 2021-07-26

**Authors:** Hua Bai, Qifang Zhang

**Affiliations:** ^1^ Department of Neurology, The Third Affiliated Hospital of Guizhou Medical University, Duyun, China; ^2^ Department of Neurology, Affiliated Hospital of Guizhou Medical University, Guiyang, China; ^3^ Medical Experimental Center of the Third Affiliated Hospital of Guizhou Medical University, Duyun, China; ^4^ Key Laboratory of Endemic and Ethnic Diseases, Ministry of Education, Guizhou Medical University, Guiyang, China; ^5^ Key Laboratory of Medical Molecular Biology, Guizhou Medical University, Guiyang, China

**Keywords:** Alzheimer’s disease, inflammasome, pathogenesis, activation, nucleotide-binding domain leucine-rich repeat and pyrin domain containing receptor protein 3

## Abstract

The nucleotide-binding domain leucine-rich repeat and pyrin domain containing receptor protein 3 (NLRP3) is an important pattern recognition receptor in human innate immunity. Activation of the NLRP3 inflammasome play a key role in the pathogenesis of Alzheimer’s disease (AD). Theories explaining activation of the NLRP3 inflammasome include the reactive oxygen species theory, the lysosomal damage theory and the mitochondrial DNA theory. The NLRP3 activation promotes occurrence of AD by producing IL-1β, IL-18 and other cytokines, and then by affecting the deposition of Aβ and tau proteins. Over-activated NLRP3 inflammasome often impair cell function and induces immune-related diseases. Some mechanisms have been found to negatively regulate activation of the NLRP3 inflammasome, which may be through receptor binding blocking mechanism, autophagy related mechanism, abnormal cytokine secretion mechanism, or interference related gene expression regulation mechanism. In this review, we summarize the possible mechanisms by which the activation of NLRP3 inflammasomes affects the pathogenesis of AD, and the recent advances in the prevention and treatment of AD by controlling the activation of NLRP3 inflammasomes. By researching the activation or inactivation of NLRP3 inflammasome, it is possible to reveal the pathogenesis of AD from a new perspective and provide a new idea for the prevention and treatment of AD.

## Introduction

With the increase in the elderly population, the incidence of Alzheimer’s disease (AD) has a trend of increasing year by year (1). AD is a neurodegenerative disease. Its main clinical manifestations are memory disorder, personality abnormality, apraxia, visual space abnormality, executive dysfunction and neuropsychiatric symptoms (2, 3). The pathogenesis of AD is mainly related to the deposition of β-amyloid protein (Aβ), the neurofibrillary tangle caused by phosphorylation of microtubule associated protein and the loss of neurons. Compared with the healthy brain, the brains of AD patients have a significant accumulation of tau protein and interacts with abnormal Aβ proteins (4). The main pathological changes in the brain of AD patients under the microscope are senile plaques, neurofibrillary tangles, vacuolar degeneration of neuronal particles, cerebral amyloid angiopathy and glial cell hyperplasia (3). The above pathological changes are closely related to the innate immune abnormalities in the brain, in which the non-specific damage of brain blood vessels and the immune inflammatory response of glial cells play important roles (5, 6).

The etiology of AD are various and hidden, and the pathogenesis of AD is complex and varied. However, the role of inflammasomes, especially the nucleotide-binding domain leucine-rich repeat and pyrin domain containing receptor protein 3 (NLRP3) inflammasome, has recently been highlighted (7–9). NLRP3 is a very important pattern recognition receptor in human innate immunity. As an indispensable component of natural immunity, the NLRP3 inflammasome plays a key role in the human immune response and onset of some immune-related diseases (10). The NLRP3 inflammasome can regulate activation of caspase-1, thereby promoting maturation and secretion of the cytokines interleukin-1 beta precursor protein (pro-IL-1β) and pro-IL-18 during immune defense (11). More and more experimental evidence show that the activation of NLRP3 inflammasome is closely related to some immune related diseases, such as gout, multiple sclerosis, diabetes, ulcerative colitis and AD (12). Recently, great progress has been made in the study of NLRP3 inflammasome associated with AD.

## Composition and Function of NLRP3 Inflammasome

The inflammasome is composed of molecular receptor protein, adapter protein and pro-caspase-1. Molecular receptor proteins include classical NLR and non-classical NLR. Adapter protein here is an apoptosis-associated speck-like protein containing cysteine aspartic proteinase recruitment domain (ASC), Generally speaking, the adapter protein is ASC. ASC is a cohesive protein of this specific inflammasome, whose amino terminal contains pyrin domain (PYD) domain and carboxyl terminal contains cysteine aspartic proteinase recruitment domain (CARD) domain. The CARD domain acts as a recruitment effector protein, which can be matched and linked with the CARD domain in the caspase-1 precursor protein (13). Different inflammasomes are formed by different molecular receptor proteins. Some inflammasomes do not require ASC (14, 15). Inflammasomes are a kind of multi-protein complex assembled by intracytoplasmic pattern recognition receptors. They recognize the foreign pathogen molecules and the host’s own danger signals, to recruit and activate the proinflammatory protease pro-caspase-1. Activated caspase-1 can enzymatically digest and chop the precursors of IL-1β and IL-18 to produce the corresponding mature cytokines (16). At present, it is certain that a variety of inflammasomes participate in the host’s defense response against pathogens, and pathogens have also evolved multiple mechanisms to inhibit the activation of inflammasome (17).

**Figure 1:**
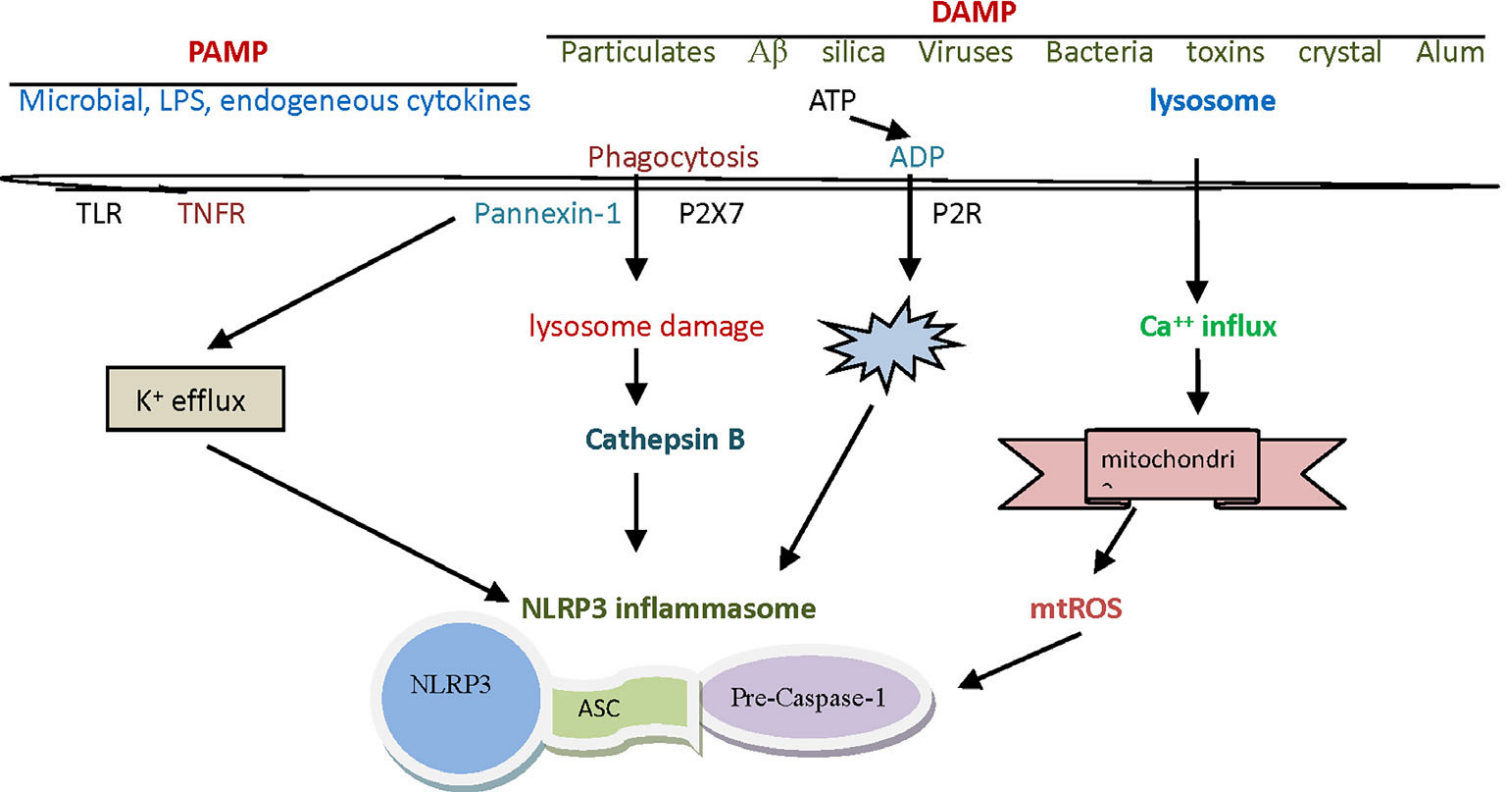
The assembly and activation of The NLRP3 inflammasome. The NLRP3 inflammasome is a complex composed of NLRP3, ASC and caspase-1 precursor proteins, in which NLRP3 protein plays a leading role. ASC is a cohesive protein of this specific inflammasome, whose amino terminal contains pyrin domain and carboxyl terminal contains CARD domain. The initiation signals of activating NLRP3 inflammasome are some internal and external activators, including a variety of PAMP and DAMP. NLRP3, nucleotide-binding domain leucine-rich repeat and pyrin domain containing receptor protein 3; ASC, apoptosis associated speck like protein containing cysteine aspartic proteinase recruitment domain; CARD, cysteine aspartic proteinase recruitment domain; PAMP, pathogen-associated molecular patterns; DAMP, danger associated molecule patterns.

The NLRP3 inflammasome is a complex composed of NLRP3, ASC and caspase-1 precursor proteins, in which NLRP3 protein plays a leading role (18). NLRP3 is a NOD like receptor protein with pattern recognition, with carboxyl terminus and leucine rich repeat domain, but lacking CARD domain. So it is not possible to bind to caspase-1 precursor protein directly. Instead, it is necessary to recruit caspase-1 precursor protein by binding to ASC through the pyrin domain at the N-terminal, and then activate caspase-1 (**Figure 1**). NLRP3 binds the adaptor ASC through PYD-PYD interactions, and ASC binds pro-caspase-1 *via* CARD-CARD domain interactions. The caspase-1 precursor protein first forms a tetramer for self-activation, followed by a heterodimer with enzymatic activity (19). Caspase-1, as an inflammasome effector protein, is able to cut the inactive IL-1β precursor protein and IL-18 precursor protein into mature IL-1β and IL-18, respectively, so as to play a variety of non-specific inflammatory roles (20). The abnormality of NLRP3 inflammasome is closely related to the onset of various refractory diseases (such as diabetes, AD, etc.) (21). Therefore, it is of great clinical significance to study the activation and function of the NLRP3 inflammasome.

## Activation of NLRP3 Inflammasome

The initiation signals of activating NLRP3 inflammasome are some internal and external activators, including a variety of pathogen-associated molecular patterns (PAMP) and danger associated molecule patterns (DAMP). The PAMP signals that can be detected by the NLRP3 inflammasome are silica, calcium pyrophosphate, sodium urate, palmitate, cholesterol, sodium A, and certain viruses. Lipopolysaccharide (LPS) is often considered a prototypic PAMP. Except for viruses, other pathogenic factors mentioned above are sometimes considered to belong to DAMP (22). It has been found that DAMP signals that can be sensed by the NLRP3 inflammasome include hypokalemia, hypercalcemia, hyperglycemia, cathepsin B, adenosine triphosphate (ATP), and reactive oxygen species (ROS), etc. (**Table 1**).

**Table 1:** The activated mode and steps of NLRP3 inflammasome and the theories that explain these activation.

	Activating molecules	Activation steps	Theories for explaining these activation
PAMP	Calcium pyrophosphate, sodium, LPS, urate, palmitate, cholesterol, etc.	Inducing inflammation, oligomerization of NLRP3, assembly of ASC and caspase-1 precursor protein.	Active oxygen theory, lysosomal damage theory, mitochondrial DNA theory
DAMP	Hypokalemia, hypercalcemia, cathepsin B, Silica, Aβ, ATP, hyperglycemia, ROS, etc.	oligomerization of NLRP3, assembly of ASC and caspase-1 precursor protein.	Active oxygen theory, mitochondrial DNA theory

Activation of the NLRP3 inflammasome by foreign signals involves complex pathophysiological and biochemical processes, and currently there are mainly three theories to explain this activation mechanism (23). The first theory is the reactive oxygen theory, which believes that reactive oxygen species (ROS) are key mediators in the regulation of NLRP3 inflammasome, and that it is associated with high concentration or high expression of catalase or reduced nicotinamide adenine dinucleoside phosphate oxidase. In normal physiology, the thioredoxin interaction protein (TXNIP) and thioredoxin (TRX) are bound to each other. When certain oxidative cell stress increases ROS, TRX oxidizes itself in order to remove ROS, TXNIP and TRX are separated (18). TXNIP was then induced to bind to NLRP3, thereby relying on ASC to recruit caspase-1 precursor protein, and finally to complete the assembly and activation of the NLRP3 inflammasome (**Figure 1**). Crystals, high glucose, ATP and other activators (activation signals) usually activate NLRP3 inflammasome in this way (24, 25). Another theory is the lysosomal damage concept, which believes that PAMP, when swallowed by macrophages, destabilizes the phagocytes, leading to lysosomal acidification and rupture, from which cathepsin B is normally released into the cytoplasm. Since cathepsin B can degrade the inhibitory protein of NLRP3, it can activate the inflammasome of NLRP3 (26).

Macrophages must first be exposed to initiating stimuli to activate the transcription factor NF-kB through toll-like receptor ligands or cytokine receptors, to up-regulate NLRP3 expression. Molecules that regulate NF-kB activity can indirectly affect NLRP3 priming. Activated NF-kB transcribes NLRP3 mRNA and then performs translation and post-translational modifications to complete the NLRP3 priming process (27). The third theory is the mitochondrial DNA theory, which believes that various PAMP **or** DAMP in the body can attack or damage the mitochondria in the body’s cells under certain conditions. Damaged mitochondria often release mitochondrial deoxyribonucleic acid (mtDNA), which activates NLRP3 inflammasome by potassium outflow or calcium influx. In this activation mode, the destruction of cell membrane by cytotoxin, ATP binding to purinergic ligand-gated ion channel 7 receptor (P2X7R), and the intervention in mitochondrial micropore structure by microbial toxin may contribute to potassium ion outflow. Calcium influx may be assisted by G-protein-coupled receptor, calcium-sensitive receptor, and pannexin-1 channels (28, 29).

Activation of the NLRP3 inflammasome usually requires two steps: first, initiation of an inflammatory response, and second, oligomerization of NLRP3 and assembly with ASC and caspase-1 precursors (30). The inflammatory response is usually the result of uninhibition and nuclear transposition of the nuclear transcription factor kappa B (NF-κB), which leads to transcription of NLRP3 itself and IL-1β. Many hormone-induced signaling pathways are reduced by the activation of NF-κB. The stimulation of LPS signals through toll-like receptor 4**/**leukocyte differentiation antigen 14 (TLR4/CD14) signaling pathway. The deubiquitination of NLRP3 is thought to depend on the role of mitochondrial ROS. The mitotic kinase NEK7 mediates the assembly and activation of NLRP3 inflammasome during cell division. NEK7 connects adjacent NLRP3 subunits and mediates the activation of NLRP3 inflammasome by interaction (31). Sharif et al. (32) reported that structural identification of NLRP3 and NEK7 was confirmed by *in vitro* and intracellular mutations. NEK7 mediates the activation of NLRP3 inflammasome by interacting with the two parts and connecting adjacent NLRP3 subunits. Ren et al. found (33) that ABRO1 promoted the activation of NLRP3 inflammasome by regulating NLRP3 dihydroylation. ABRO1 as a scaffold for the interaction between NLRP3 and breast cancer susceptibility gene complex subunit protein 3 (BRCC3), acting synergistically with BRCC3 promotes the inflammatory activation of NLRP3 by regulating the deamination of NLRP3. In addition, blocking the activity of ABRO1 can prevent the activation of the relevant inflammatory cells.

Changes in potassium and calcium ion concentrations can affect activation of the NLRP3 inflammasome. The classical NLRP3 activation pathway can lead to the increase of intracellular calcium ion concentration, enhancing the intracellular calpain activity. When membrane potential is depolarized by high concentration of potassium ions in extracellular solution, or is hyperpolarized by small molecule compounds, calpain activity can be decreased and activation of NLRP3 inflammasome can be blocked (13). Thus, it was confirmed that the continuous changes of membrane potential inhibited the activation of calpain, which was thought as a new molecular mechanism of calpain regulating the assembly of NLRP3 inflammasome under resting membrane potential (34). In addition, the treatment of high concentration of potassium ions outside the cell caused the continuous depolarization of cell membrane potential by obstructing the outflow of potassium ions, which have dramatic impact on intracellular signal transduction and activity of biological macromolecules. Although NLRP3 inflammasome can be activated during the early stages of crystal stimulation without decreasing intracellular potassium concentration, sometimes the initiation of NLRP3 inflammasome activation signaling is not affected by the concentration of potassium efflux (35).

## Negative Regulation of NLRP3 Inflammasome Activation

**Figure 2:**
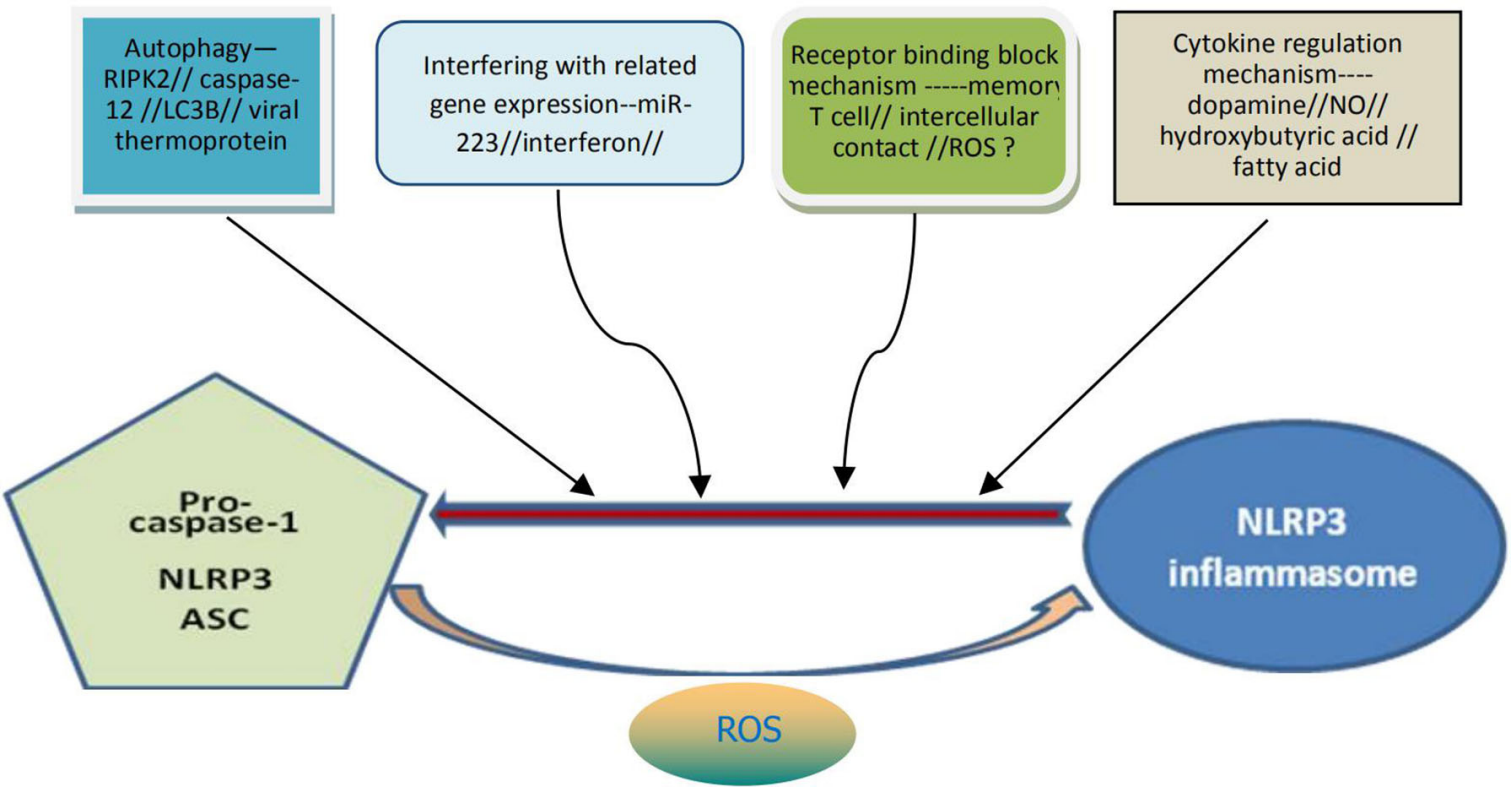
Possible specific mechanisms for negative regulation of NLRP3 inflammasome activation. The activation of NLRP3 inflammasome can be negatively regulated through at least four pathways: the mechanism of autophagy, the mechanism by interfering with the related genes expression, the mechanism of receptor binding and blocking, the mechanism from cytokine regulation. It can also be understood that the above four pathways contribute to the decomposition and destruction of NLRP3 inflammasome. ROS may be dual and generally contribute to the formation and activation of NLRP3 inflammasome. NLRP3, nucleotide-binding domain leucine-rich repeat and pyrin domain containing receptor protein 3; ROS, reactive oxygen species.

Proper activation of NLRP3 inflammasome may be beneficial to the human body under certain conditions as it can help to resist exogenous microbial infection and endogenous cell damage. Overactivation of the inflammasome, however, is usually detrimental to cellular and body health. At present, it has also been found (36) that there are various mechanisms in cells of the body for the negative regulation of the activation of NLRP3 inflammasome, which may function through the mechanism of receptor binding and blocking, the mechanism of autophagy, or by interfering with the expression mechanism of related genes (**Figure 2**). For example, mir-223 negatively regulates the activation of NLRP3 inflammasomes by inhibiting the expression of NLRP3 gene at the transcriptional level. T cells and interferon inhibit activation of the NLRP3 inflammasomes by down-regulating the expression of P2X7R and interfering with signal transduction and transcriptional activation factor (STAT) signaling pathways, respectively. Bai et al. (37) found that hydrogen peroxide treatment activated NLRP3 inflammasome and reduced signal transduction and phosphorylation of STAT serine 727 *in vitro* cultured cells. Preconditioning with BAPTA-AM inhibited the activation of NLRP3 inflammasome induced by hydrogen peroxide. Downregulation of STAT gene expression may enhance NLRP3-mediated oxidative stress, a process independent of calcium signaling. In addition, dopamine, nitric oxide, β-hydroxybutyric acid and unsaturated fatty acids can inhibit activation of the NLRP3 inflammasome. The increase of autophagy protein LC3B will lead to the decrease of ROS level, thus inhibiting activation of the NLRP3 inflammasome (**Figure 1**). Viral thermal proteins can interfere with the binding between ASC and NLRP3 by PYD-PYD, thus negatively regulating the assembly and activation of the NLRP3 inflammasome. Caspase-12 also negatively regulates activation of the NLRP3 inflammasome through CARD-CARD competitively binding ASC (38).

Mitochondria play an unique role in the activation and regulation of the NLRP3 inflammasome. Localization of NLRP3 in mitochondria is critical for activation of the NLRP3 inflammasomes, and mitochondrial adaptor proteins are necessary for optimal activation of the NLRP3 inflammasome. Mitochondrial antiviral signaling protein not only mediates the response of type I interferon against virus, but also as a bridge for mitochondrial regulation of the NLRP3 inflammasome (39). Actin-dependent mitochondrial ASC transfer is mediated by acetylation, which causes the mitochondrial ASC to interact with the endoplasmic reticulum NLRP3. Autophagy related proteins can maintain mitochondrial integrity and inhibit the NLRP3 inflammasome activation. During influenza virus infection, RIPK2 protein can inhibit the activation of NLRP3 inflammasome by promoting mitochondrial autophagy (40).

The regulatory effect of ROS on the NLRP3 inflammasome remains controversial. Although some experiments have proved that ROS can mediate activation of the NLRP3 inflammasome, others have found that ROS can only promote the activation of NLRP3 protein, but have no effect on the activation of the NLRP3 inflammasome (41). In the resting state, the expression level of NLRP3 in cells is very low, so the activated NF-κB function is needed to transcribed NLRP3 mRNA, and then the translation and post-translation modification can be carried out. In addition, lysosomes, as an organelle for the decomposition of various macromolecules in eukaryotic cells, also participate in the regulation of activation of the NLRP3 inflammasome. These phagocytic particles may promote the activation of NLRP3 inflammasome by acting directly with inflammasome related proteins, and may also produce some ROS, which play a negative feedback regulating role in the activation of NLRP3 (42).

There are also very unique ways in which NLRP3 inflammasome activation is negatively modulated. Type I interferon (IFN) inhibits the production of interleukin-1 and the activation of inflammasomes. IFN inhibits the production of IL-1β through two different mechanisms. The IFN signaling pathway inhibits the activity of NLRP1 and NLRP3 inflammasomes through the STAT1 transcription factor, thereby inhibiting caspase-1-dependent IL-1β maturation. In NLRP3 dependent peritonitis models, effector T cells reduced neutrophils recruitment in an antigen-dependent manner, demonstrating that T cells inhibit innate immune response by inhibiting NLRP3 inflammasomes (43).

## Some Clues to the Onset of AD After the Activation of NLRP3 Inflammasome

When the NLRP3 inflammasome is activated, it can promote the onset of AD in two ways. First, it can regulate IL-1β, which produces neurotoxins that cause degeneration of neurons. Second, it can cause an increase in Aβ deposition, thus inducing Aβ self-perpetuating positive feedback loop, which eventually leads to the development of AD. The exposed complex of ASC with Aβ can amplify proinflammatory response, lead to inflammatory cell death, release functional ASC and induce a vicious cycle of feedforward stimulation.

NLRP3 inflammasome is activated not only by fibrillar Aβ aggregates, but also by lower molecular weight Aβ oligomers and protofibrils (44). It has been found that high expression of NLRP3 and caspase-1 genes was conducive to the formation of aging lesions in the brains of amyloid precursor protein/presenilin-1 (APP/PS-1) transgenic mice (45). By reducing the activity of caspase-1 and IL-1β in the brain, it help clear Aβ. By reducing the activity of caspase-1 and IL-1β in the brain, it is helpful to eliminate Aβ. In NLRP3 knockout mice, Aβ deposition was reduced and spatial memory was improved (9). On the other hand, the activation of the NLRP3 inflammasome can reduce the phagocytosis of microglia on Aβ, thus increasing Aβ deposition and promoting the occurrence and development of AD lesions (46).

**Figure 3:**
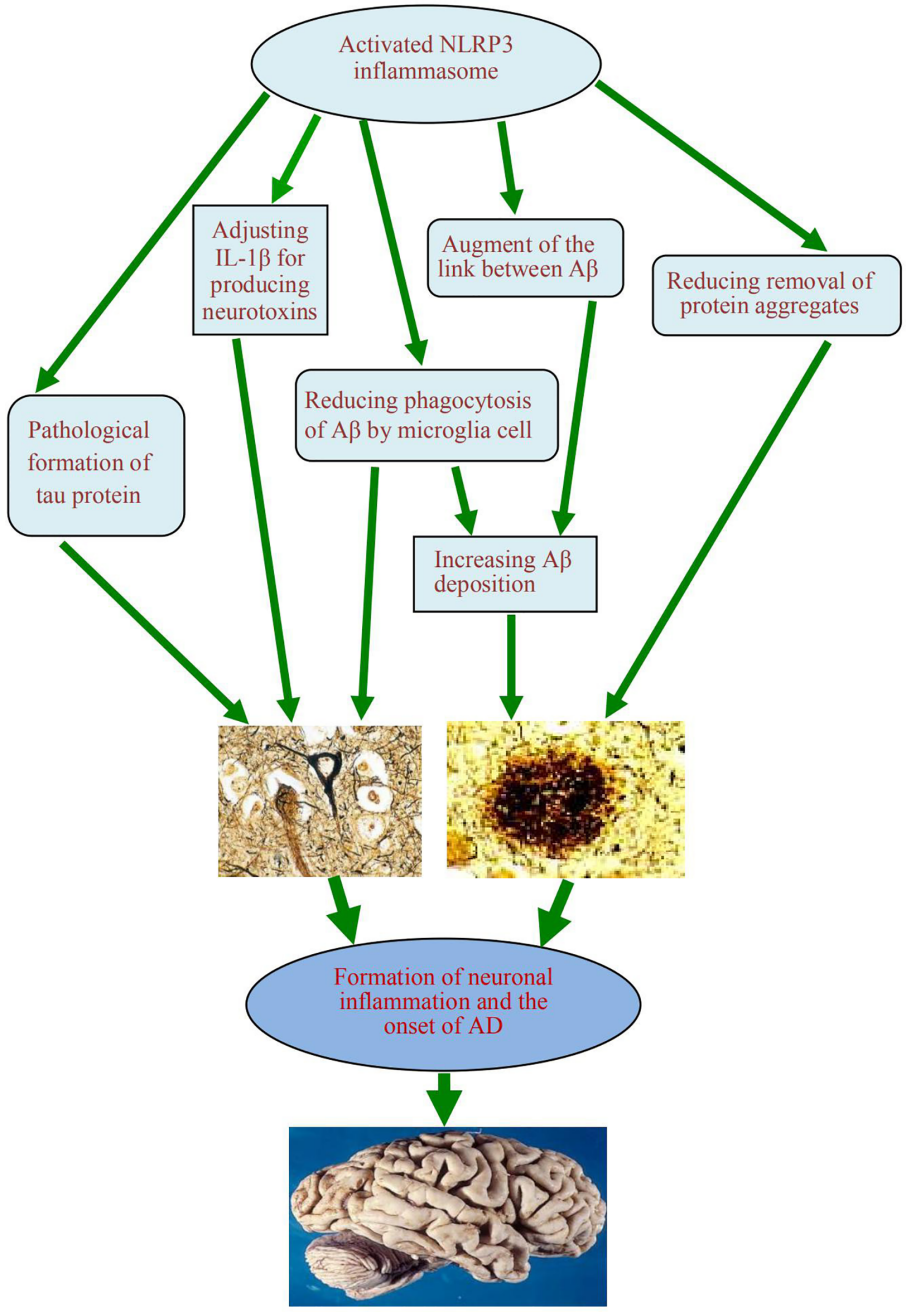
A schematic diagram of the association between activation of NLRP3 inflammasome and occurrence of AD. The activated NLRP3 inflammasome contributes to the formation of chronic neuroinflammation and the pathogenesis of Alzheimer’s disease, which play the role by regulating the production of neurotoxin IL-1β, reducing the phagocytosis of Aβ from microglia, augmenting the link between Aβ and Aβ, reducing the clearance of some excess protein aggregates, and promoting the pathological formation of Tau protein. NLRP3, nucleotide-binding domain leucine-rich repeat and pyrin domain containing receptor protein 3; AD, Alzheimer’s disease; IL-1β, interleukin 1β; Aβ, β-amyloid protein.

Using the autopsy brains of human AD patients and age matched non AD patients as the control materials, the relationship between the NLRP3 inflammasome and autophagy lysosome labeled A0205 protein, or p-tau protein, or glial maturation factor (GMF) was analyzed by immunohistochemistry, Ahmed et al. found (47) that the neuroinflammatory effect promoted by the NLRP3 inflammasome can be amplified and regulated by GMF, and further reduce the clearance of protein aggregates mediated by autophagy signaling pathway. Saresella et al. (48) found that in peripheral monocytes of patients diagnosed with AD, there was evidence of activation of both the NLRP3 inflammasome and the NLRP1 inflammasome after LPS or Aβ stimulation. They believed that the migration of peripheral monocytes in the blood-brain barrier was likely to be an important factor leading to AD neuroinflammation. Nucleoside reverse transcriptase inhibitors can inhibit activation of these inflammatory factors. Activation of the NLRP3 inflammasome can also promote the pathological formation of tau protein, which is conducive to onset of AD (8). However, the function loss of the NLRP3 inflammasome can reduce the hyperphosphorylation and aggregation of tau by regulating tau kinase and phosphorylase. This confirmed the role of the activation of the NLRP3 inflammasome in microglia for the pathogenesis of tau related diseases, and supported the Aβ cascade hypothesis in the pathogenesis of AD (49). In addition, the role of neurofibrillary tangles in the downstream development of Aβ-induced microglia activation was also demonstrated (**Figure 3**).

NLRP3 inflammasome can be activated by a variety of dangerous signals, including pathogenic microorganisms and metabolic disorders. Several studies revealed that in the brains of AD patients, neuroinflammatory plaques can secrete neurotoxic factors, recruit microglia that engulf the aggregate Aβ, and secrete chemically pro-inflammatory molecules, thereby further promoting the damage of surrounding tissues and enhancing the neurotoxic effects of Aβ (50). The deposition of Aβ and the activation of the NLRP3 inflammasome may be mutually causal. The activated intracellular NLRP3 inflammasome can induce M1 phenotype activation of microglia and result in deposition of Aβ, and increased cognitive impairment in AD mouse models (21). In contrast, in microglia with specific functional impairment of NLRP3 inflammasome, these cells are biased toward the M2 phenotype, thereby reducing extracellular Aβ deposition, protecting nerve cells from synaptic dysfunction, and alleviating cognitive decline in the brain (51).

Thioredoxin interaction protein (TXNIP) is an endogenous regulator of REDOX/glucose-induced stress and inflammation. The components of TXNIP and NLRP3 were analyzed by Western Blot, real-time PCR and immunohistochemistry in the cerebral cortex sample from human autopsy, showing that TXNIP protein and mRNA in the cortex of AD were significantly up-regulated. Using double immunofluorescence staining, TXNIP and IL-1β were co-located in the vicinity of Aβ plaque and p-tau protein. These results suggest a strong association between TXNIP overexpression and the pathogenesis of AD. It is speculated that the activation of NLRP3 inflammasome in human AD brain may promote the occurrence of neurodegeneration. TXNIP has potential as a molecular link between chronic immune inflammation and AD, and is expected to be a new therapeutic target in the future (52, 53).

In fact, the elevated IL-1β level in the brain of patients with AD and Lewy body pathologies had been reported as early as ten years ago, it may be the end product of some inflammasome activation (54, 55). After treating microglia cells with Aβ, the expression of IL-1β was significantly decreased in the treatment group with lysosomal inhibitor cytochalasin D or cathepsin B inhibitor (46). It is suggested that Aβ can activate the NLRP3 inflammasome by lysosomal rupture pathway to induce the high expression of IL-1β, thus promoting the occurrence and development of AD. After their phagocytosis, Aβ fibrils are localized in intracellular lysosomes, and damage to the membranes of these lysosomes results in the release of cathepsin B into the cytoplasmic sol. A pattern recognition receptor cluster of differentiation 36 (CD36) mediated endocytosis pathway that coordinates the transformation of these soluble ligands into crystals or fibers in cells, leading to lysosomal destruction and activation of the NLRP3 inflammasome (38). It should be pointed out that the activation of NLRP3 inflammasome is not always harmful. At least in the early stage, the activation of NLRP3 inflammasome in some tissue cells may help to reduce the damage, or the activation of such inflammasome may have a protective effect on the organism (56).

In order to evaluate the role of NLRP3 inflammasome in the pathogenesis of AD, Heneka et al. (57) used NLRP3 gene knockout mice to hybridize with APP/PS1 transgenic mice. The results showed that there was a lack of caspase cleavage in the brains of the hybrid mice, and that the total IL-1β level was similar to that of wild-type mice, suggesting NLRP3 is important for the pathogenesis of AD. One of the components of the NLRP3 inflammasome, apoptosis-associated speck-like protein containing CARD, was detected by immunohistochemistry. Spot formation of microglia was detected in activated microglia from transgenic APP/PS1 mice with positive Iba1 marker, a change consistent with activation of inflammasomes. Wild-type mice, NLRP3 knockout mice, AD mice, and APP/PS1/NLRP3 hybrid knockout mice were tested and compared by water maze test. The results showed that APP/PS1 mice showed severe spatial memory formation defects, while APP/PS1/NLRP3 hybrid knockout mice showed reduction of spatial and memory impairment to a large extent. By examining the plasticity of synapses in the hippocampus of mice by detecting long-term potentiation (LTP), the authors also found that mice with defects in NLRP3 or caspase-1 completely blocked LTP inhibition (29). As memory is encoded by the plasticity of synapses, LTP is considered to be positively correlated with memory and inversely correlated with the severity of AD. The above experiments confirmed from another perspective that the activation of the NLRP3 inflammasome can promote the onset of AD.

## Control of the Onset of AD by Inhibiting the Activation of NLRP3 Inflammasome

Inhibiting the activation of NLRP3 inflammasome has a great prospect for the prevention and treatment of AD. β-hydroxybutyrate (BHB) is a ketone which can effectively cross the blood-brain barrier and inhibit the activation of NLRP3 inflammasome in human monocytes (58). Some experiments have found that the level of BHB in the blood of AD patients is obviously low (59). When the level of BHB in the blood is increased, the cognitive function of AD patients is improved (60). Shippy et al. found (61) that the level of BHB in brain tissue and red blood cell samples of AD patients was significantly lower than that of non ad control group; BHB inhibited NLRP3 inflammasome in bone marrow-derived macrophages (BMDM) by reducing the levels of IL-1 β and caspase-1. 5xFAD mice which is an APP/PS1 transgenic AD mouse model with 5 family gene mutations was used here, BHB treatment showed significantly reducing the number and volume of pathological plaques in the cerebral cortex of AD mice. Consistent with the reduction of NLRP3 inflammasome activation, the apoptosis related spot like protein containing caspase recruitment domain in BHB treated 5xFAD mice was significantly reduced. It suggests that BHB or BH derivatives are promising drugs for the prevention and treatment of AD (61).

More and more evidence shows that neuroinflammation is an important factor in the onset of Alzheimer’s disease (AD). IL-1β may be the main inducer of this inflammation. IL-1β expression is upregulated in the brain of AD patients, and inhibition of the activation of NLRP3 inflammasome can improve the behavioral abnormalities and synaptic pathological phenotype in AD mice (39). Dapansutrile (OLT1177) is a specific inhibitor of NLRP3. Lonnemann et al. (62) used OLT1177 to inhibit the activation of NLRP3 inflammasome and thus improve the function of AD mice. Their experiment showed: 6 months when the size of APP/PS1 mice were fed standard chow and add OLT1177 food after 3 months, can improve the learning and memory ability of mice, normalize AD metabolic markers in plasma, and restore the synaptic plasticity, reduce the number of pathological plaques in the cerebral cortex, and reducing the brain glial cell activity. In the future, OLT1177 is expected to be developed as a new drug for the treatment of AD. Diacetyl-p-phenylenediamine is a synthetic chemical containing benzene ring, which has the ability to regulate the function of microglia, inhibit neuronal inflammation, and promote the elimination and alleviation of cognitive impairment in AD brain. In transgenic AD mouse model, diacetyl-p-phenylenediamine can promote microglia phagocytosis and improve the cognitive function of mice by affecting NF-κB signaling pathway and inhibiting the expression of NLRP3 Dysfunction (63).

Some herbs or their extract can inhibit the activation of NLRP3 inflammasome and show the effect of prevention and treatment of AD (64–66). Radix Scrophulariae (RS) is a kind of herb that has anti-oxidation, anti-inflammatory, anti-allergic, and anti-cancer features. Kim et al. (67) found that IL-1β was effectively reduced after treatment with RS, along with the down-regulation of NLRP3 protein, and inhibition of caspase-1 activity in 5XFAD mice. Two months after treatment with RS, Aβ and plaque bulk were obviously decreased in the hippocampus of the brains of 5XFAD mice after treating with Radix Scrophulariae. In addition, RS can regulate neuroinflammation and down-regulate the expression of BACE1 protein in 5XFAD mice. BACE1 is a protein that can increase Aβ formation. It has been found that fresh coconut oil shows potential neuroprotective effects by inhibiting the activation of NLRP3 inflammasomes, mainly by antagonizing Aβ-induced neurotoxicity (68). Pterostilbene is a compound extracted from grapes and strawberries and can inhibits Aβ-induced neuroinflammation in a microglia cell line by inactivating the NLRP3 inflammasome (69).

Neuroinflammation and autophagy dysfunction are involved in the pathological process of AD, and the autophagy-lysosomal pathway is a specific molecular mechanism that modulates Aβ-induced activation of NLRP3-caspase-1 inflammasome. Hong et al. found that progesterone (PG) exhibited a neuroprotective effect by inhibiting Aβ-induced activation of NLRP3 inflammasomes by enhancing autophagy in astrocytes (70). Stavudine (D4T) is a nucleoside reverse transcriptase inhibitor, which can reduce NLRP3 assembly as well as IL-18 and caspase-1 production, but did not affect IL-1β production and TREM2 expression. In an *in vitro* AD model of Aβ-driven neuroinflammation, D4T reduces NLRP3 inflammasome-associated inflammation and stimulates Aβ autophagy by macrophages (58). 2,3,5,4 ‘-Tetrahydroxy stilbene-2-O-β-D-glucoside (TSG) is a promising chemical for drug development in the treatment of AD, which has been shown to be beneficial in reducing cognitive function in animal models of AD. TSG can effectively alleviate LPS-induced neuroinflammatory response by inhibiting the NLRP3 signaling pathway of microglia and neurons. Meanwhile, it can inhibit the activity of NLRP3 inflammasome and regulate mitochondrial autophagy, especially in microglia cells. In addition, TSG also promoted autophagy involved in AMPK/PINK1/Parkin signaling pathway, suggesting a neuroprotective effect dependent on PINK1. When PINK1 is deficient, autophagy is inhibited. Knocking out PINK1 or Parkin genes *via* the CRISPR/Cas9 system may disrupt the neuroprotective effects of TSG (71).

## Conclusions

Chronic inflammation during AD is mainly mediated by abnormal immune function of brain cells, which is generally believed to be caused by the abnormal accumulation of some proteins in microglia, mainly related to the activation of NLRP3 inflammasomes (72). As for the pathogenesis of AD, there have been the theory of β-amyloid protein, the theory of abnormal neurotransmitters, the theory of abnormal Tau protein metabolism and the theory of nerve cell apoptosis. Recently, the antimicrobial protection theory of AD disease has attracted attention. This theory holds that Aβ is a highly conserved innate immune effector molecule, and the oligomerization of Aβ protein and the production of β-amyloid are important innate immune pathways that mediate pathogen capture and protect infection. The β-amyloid deposition mimics an innate immune response to perceived immune stimulation, and since the brains of some AD-susceptible patients may sometimes have microbial infections, Aβ first traps and neutralizes invading pathogens with β-amyloid, acting as an antimicrobial peptide-like protein (73). Aβ fibrosis drives neuroinflammatory pathways, helps fight infection and clears deposition of harmful pathogens. In the pathogenesis of AD, the abnormal pathophysiological behavior pattern of Aβ is manifested as an innate immune response disorder, leading to persistent chronic immune inflammation and neurodegenerative diseases (74). Theory of neuroinflammation induced by activation of NLRP3 inflammasome suggests that congenital immune disorders and neuroinflammation play an important role in the pathogenesis of AD (75). NLRP3 inflammasome activators promote neuroinflammation and senile plaque formation through a variety of pathways. NLRP3 inflammasomes usually remains activation and regulate the excretion of harmful inflammatory molecules in the brains of AD patients. Inhibition of the activation of NLRP3 inflammasome may be an important way to treat AD patients in the future.

## Author Contributions

HB designed and wrote the manuscript. QZ discussed and revised the manuscript. All authors contributed to the article and approved the submitted version.

## Funding

This work was supported by National Natural Science Foundation of China (to BH), No:32060182, and the grant of Science and Technology Support Plan of Guizhou Province in China, No. QianKeHe-Zhicheng [2020]4Y129 (to BH).

## Conflict of Interest

The authors declare that the research was conducted in the absence of any commercial or financial relationships that could be construed as a potential conflict of interest.

## Publisher’s Note

All claims expressed in this article are solely those of the authors and do not necessarily represent those of their affiliated organizations, or those of the publisher, the editors and the reviewers. Any product that may be evaluated in this article, or claim that may be made by its manufacturer, is not guaranteed or endorsed by the publisher.

## Abbreviations

Aβ, β-amyloid protein; ABRO1, Abraxas Brother 1; AD, Alzheimer’s disease; APP, Amyloid precursor protein; ASC, Apoptosis associated speck like protein containing cysteine aspartic proteinase recruitment domain; ATP, Adenosine triphosphate; BRCC3, Breast cancer susceptibility gene complex subunit protein 3; CARD, Cysteine aspartic proteinase recruitment domain; CD36, Cluster Of Differentiation 36; EPM, Endogenous pathogenic molecules; GMF, Glial mature factor; D4T, 2′,3′-Didehydro-3′-deoxythymidine (Stavudine); DAMP, danger associated molecule patterns; IL-18, Interleukin 18; LPS, Lipopolysaccharide; LTP, Long term potentiation; MG132, Carbobenzoxy-Leu-Leu-leucinal; mtDNA, Mitochondrial deoxyribonucleic acid; NEK 7, never in mitosis gene A related Kinase 7; NF-κB, Nuclear transcription factor kappa B; NLR, Nucleotide-conjugated oligomeric domain-like receptor; NLRC4, Nucleotide-conjugated oligomeric domain-like receptor family cysteine aspartic proteinase recruitment domain domain-containing 4; NLRP3:Nucleotide-binding domain leucine-rich repeat and pyrin domain containing receptor protein 3; NOD, Nucleotide-conjugated oligomeric domain; P2X7R, Purinergic ligand-gated ion channel 7 receptor; PAMP, Pathogen-associated molecular pattern; PG, Progesterone; pro-IL-1β, Interleukin-1 beta precursor protein; PS1, Presenilin-1; PYD, pyrimidine nucleoside; ROS, Reactive oxygen species; RS, Radix Scrophulariae; STAT, Signal transduction and transcriptional activation factor; TRX, Thioredoxin; TXNIP, Thioredoxin interaction protein.
